# 

**Published:** 1913-12-06

**Authors:** 


### CONTENTS.

| | PAGE |
|---|---|
| The National Medical Union ... ... ... | 239 |
| The London Ambulance Scheme ... ... ... | 240 |
| Hospital and Institutional News, including— | 241 |
| Attendants' Reduced Hours at Croydon—Phthisical Problem in London Infirmaries—Mental Patients and the Panel System—Famous Pictures for a Cork Hospital—Medical Canvassing for Insured Patients—The British Hospital for Mental Disorders Again. | |
| Common Errors in Diagnosis: Sprains and Fractures ... ... ... ... ... ... ... | 247 |
| By ALBERT CARLESS, M.S., F.R.C.S. | |
| The Evolution of the Isolation Hospital ... | 249 |
| By the late H. FRANKLIN PARSONS, M.D., D.P.H. | |
| Oxford and Modern Medicine ... ... ... | 251 |
| The Radcliffe Infirmary, Oxford: Its Re-creation Completed ... ... ... ... ... | 251 |
| Report on the East Suffolk and Ipswich Hospital ... ... ... ... ... ... ... | 253 |
| By Sir HENRY BURDETT, K.C.B., K.C.V.O. | |
| What is Wrong with St. George's Hospital? | 256 |
| Important Conference of Non-Panel Practitioners ... ... ... ... ... ... | 257 |
| National Insurance Act: The Prospect of the First Valuation ... ... ... ... ... | 263 |
| The Institutional Library ... ... ... ... | 265 |
| Massage in England in 1913 ... ... ... | 267 |
| Within the Hospitals: The London ... ... | 268 |
| The Editor's Letter-Box ... ... ... ... | 262 |
| Worn-out Hospital Officials—Licensed Homes for Inebriates. | |
| Appointments ... ... ... ... ... ... | 270 |
| Personalia ... ... ... ... ... ... | 270 |
| London Healing Wells ... ... ... ... | 270 |
| The Institutional Worker ... ... (Suppl.) | 1 |
| Our Bureau of Information ... ... ,, | 2 |
| The Book Market ... ... ... ,, | 3 |

